# Aspirin for metal stent in malignant distal common bile duct obstruction (AIMS): study protocol for a multicenter randomized controlled trial

**DOI:** 10.1186/s13063-020-4083-z

**Published:** 2020-01-30

**Authors:** Jin Ho Choi, Woo Hyun Paik, Min Su You, Kyong Joo Lee, Young Hoon Choi, Bang-sup Shin, Sang Hyub Lee, Ji Kon Ryu, Yong-Tae Kim

**Affiliations:** 1 Division of Gastroenterology, Department of Internal Medicine and Liver Research Institute, Seoul National University College of Medicine, Seoul, South Korea; 2 Department of Internal Medicine, Yonsei University Wonju College of Medicine, Wonju, South Korea

**Keywords:** Endoscopic retrograde cholangiopancreatography, Self-expandable metallic stents, Randomized controlled trial, Aspirin

## Abstract

**Background:**

Endoscopic retrograde biliary drainage (ERBD) is the treatment of choice for patients with malignant distal common bile duct (CBD) obstruction. Self-expandable metal stents (SEMS), which are commonly used in unresectable cases, have many clinical advantages, including longer stent patency. Although the expected patency of SEMS is around 8 months, it has recently been reported that the duration of SEMS’ patency in patients using aspirin is prolonged. Our study, therefore, aims to investigate the effect of aspirin on SEMS’ patency.

**Methods/design:**

This is an investigator-initiated, prospective, multicenter, double-blind, randomized placebo-controlled trial that will be conducted from November 2017 in four tertiary centers in South Korea. We intend to include in our study 184 adult (aged ≥ 20 years) patients with malignant distal CBD obstruction for whom ERBD with SEMS was successfully performed. The patients will be randomly allocated to two groups, which will comprise patients who have either taken 100 mg aspirin or a placebo for 6 months after index ERBD. The primary outcome will be the rate of stent dysfunction, and the secondary outcomes will be the duration of patency, the rate of reintervention, and the occurrence of adverse events.

**Discussion:**

The aspirin for metal stents in malignant distal common bile duct obstruction (AIMS) study should determine the efficacy of aspirin in maintaining metal-stent patency in patients with malignant distal CBD obstructive.

**Trial registration:**

ClinicalTrials.gov, ID: NCT03279809. Registered on 5 September 2017.

## Background

Endoscopic biliary drainage for malignant distal common bile duct (CBD) obstruction is the modality of choice that could resolve multiple clinical problems for patients with conditions such as jaundice, pain, sepsis, and organ failure. Several studies, including a meta-analysis, have reported that self-expandable metal stents (SEMS) have advantages over plastic stents in terms of stent patency, adverse events, revisions, and the survival of patients with malignant biliary obstruction [1–7]. It is important to maintain stent patency because cholangitis may occur due to stent dysfunction, which may affect patients’ prognoses.

SEMS do not maintain patency for long periods within an overall life span because the stent may become obstructed due to, predominantly, tumor ingrowth or overgrowth, sludge deposition, biofilm formation, or epithelial hyperplasia [8–10]. Some of these factors may be affected by aspirin when considering the pharmacological mechanisms of aspirin. Aspirin affects the cyclooxygenase pathway and prostaglandin production, which suggests that it has the potential to reduce mucin secretion and inhibit gallstone formation [11]. However, the use of systemic drugs, including ursodeoxycholic acid (UDCA) and antibiotics, to effectively prevent stent dysfunction, has not been reported [12].

Lee et al. found experimental evidence supporting the inhibitory effect of aspirin on glycoprotein secretion and crystal and stone formation in dogs [13]. It has also been reported that aspirin decreases the viscosity of bile [14, 15]. A study by Rhodes et al. found that treating patients with aspirin reduced the production of mucin in the gallbladder compared with those not treated with aspirin [16], while Sterling et al. found that the concentration of gallbladder mucin was significantly lower in chronic non-steroidal anti-inflammatory drug users [17]. However, the results of these studies regarding the true effects of aspirin were unclear. Paul et al. reported that UDCA had a marked effect on inhibiting gallstone formation, but aspirin had no significant effect [18]. Sahlin et al. advocated that the degree of stone formation does not change according to the protein content of bile and presumed that only UDCA changes the constitution of bile because the protein composition of bile was only different in the group treated with UDCA in their study, as in the case of aspirin and chenodeoxycholic acid [19].

The results of a recent large retrospective cohort study conducted by Jang et al. showed that SEMS’ occlusion in malignant distal CBD obstruction was significantly less likely among patients taking aspirin [11]. Importantly, their study suggested that the use of systemic drugs can improve stent patency. This is contrary to previous studies that have mostly focused on the type, shape, and functional aspects of stents. In this study, we aim to determine whether aspirin use has a positive effect on the patency of SEMS in unresectable, malignant, distal CBD obstruction.

## Methods/design

**Fig. 1:**
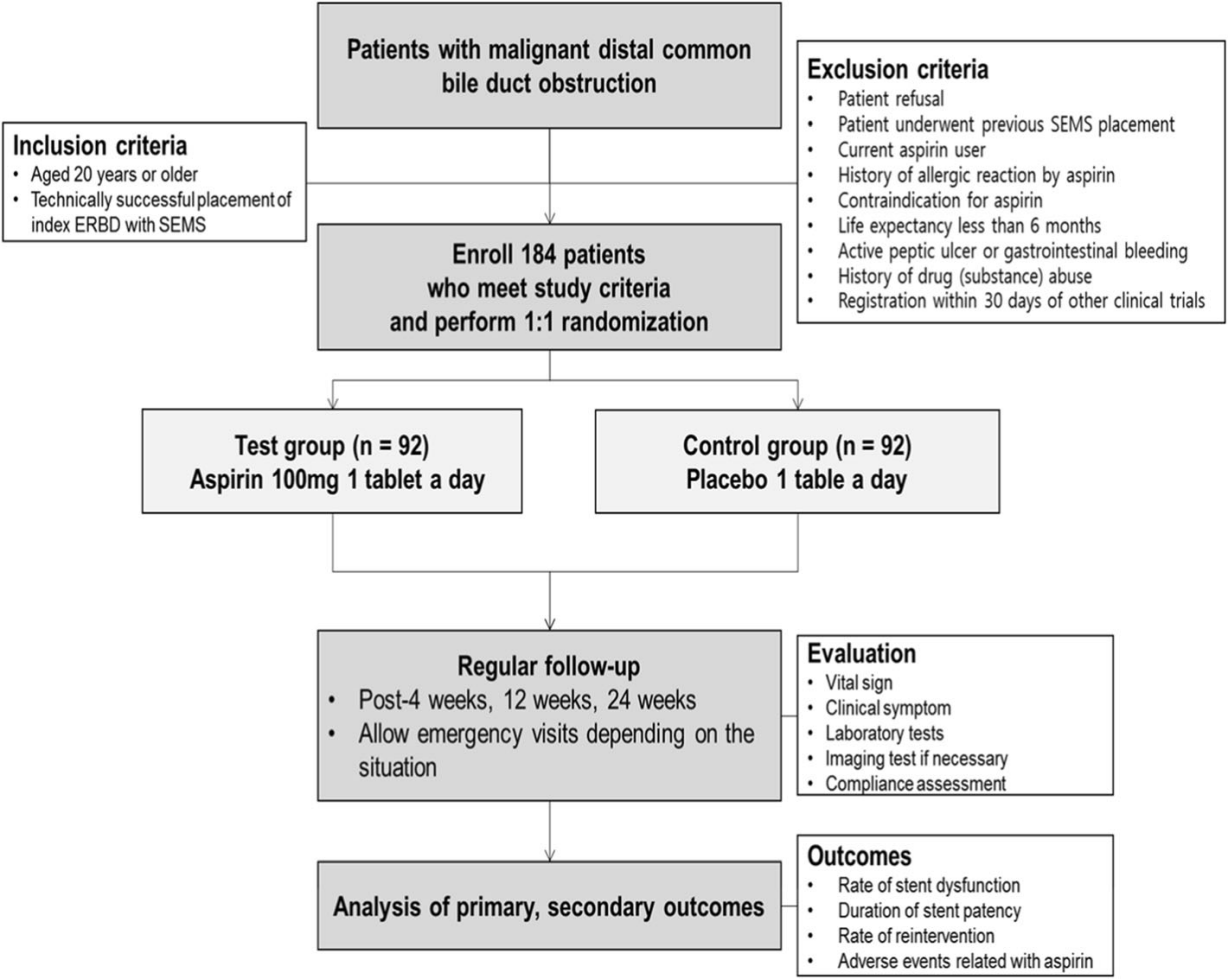
Overall scheme of the aspirin for metal stents in malignant distal common bile duct obstruction (AIMS) study

**Fig. 2:**
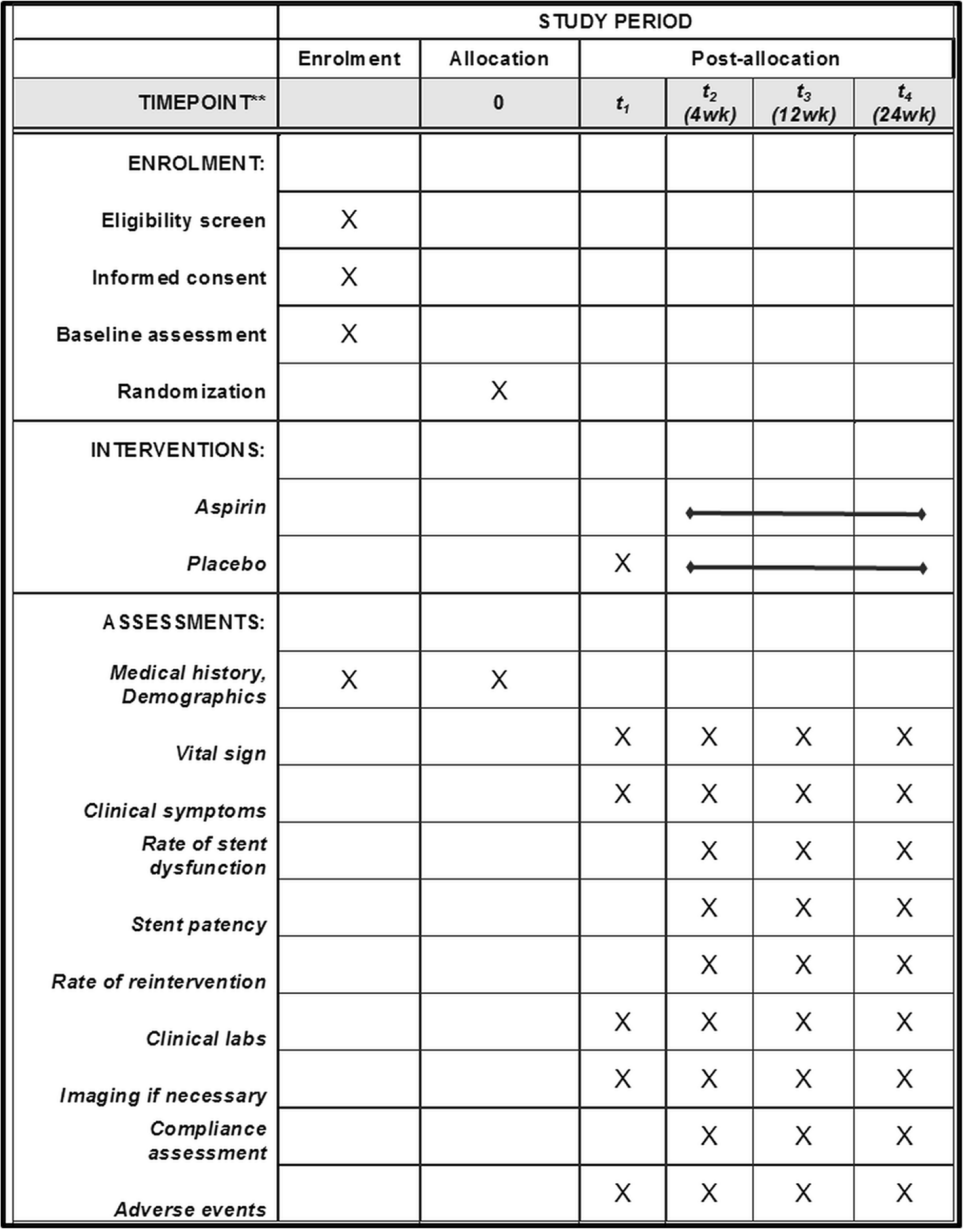
Schedule of enrollment and assessments

Our study on aspirin for metal stents in malignant distal common bile duct obstruction (AIMS) is an investigator-initiated, randomized, multicenter, double-blinded, placebo-controlled, prospective comparative study. We will perform the study in accordance with the common guidelines for clinical trials in accordance with the Declaration of Helsinki and International Conference on Harmonization and World Health Organization Good Clinical Practice (ICH-GCP) standards. This study protocol was approved by the Institutional Review Board of Seoul National University Hospital, South Korea (IRB No. H-1707-161-874). This trial was registered with ClinicalTrials.gov (ID: NCT03279809) on 5 September 2017. Figure 1 shows the overall scheme for the AIMS study and Fig. 2 the Standard Protocol Items: Recommendations for Interventional Trials (SPIRIT) Figure and Additional file 1 the SPIRIT Checklist.

### Population

Patients who need endoscopic biliary drainage for malignant distal CBD obstruction, and who have been referred to tertiary centers, will be recruited for the trial. The target sample size is 184 subjects in the tertiary referral centers in South Korea (i.e., Seoul National University Hospital, Wonju Severance Christian Hospital, Myongji Hospital, and Yongin Severance Hospital). Our study will be informed to patients who are considered eligible for the trial. All the eligible candidates will undergo an interview and receive more detailed information regarding the study, the intervention, and the two ways to manage index SEMS’ placement for malignant distal CBD obstruction. The candidates will then receive either aspirin or a placebo in a double-blind manner since evidence on the effect of aspirin for SEMS’ maintenance is unclear. The participants’ written informed consent will be obtained and archived securely in document form. The participants will be provided with comprehensive information regarding the study aim, intervention, and potential risks and benefits, and will be able to withdraw from the study at any time without consequence. The trial will commence from November 2017 and conclude 6 months after the last patient enrollment. All the recruitment procedures will be recorded in a log file.

### Inclusion criteria

The participants meeting the following criteria will be included:

1. Aged 20 years or older
2. Have unresectable, malignant distal CBD obstruction
3. Technically successful placement of the index ERBD with a SEMS

### Exclusion criteria

Participants meeting one or more of the following criteria will be excluded from the study:

1. Patient refusal
2. Patient underwent previous SEMS’ placement
3. Current aspirin user
4. History of allergic reaction to aspirin
5. Contraindication to aspirin
6. Life expectancy less than 6 months
7. Active peptic ulcer or gastrointestinal bleeding
8. History of drug (substance) abuse
9. Registration for other clinical trials within 30 days

### Randomization, blinding, and treatment allocation

The randomization lists will be generated using Random Allocation Software version 1.0.0 (free software developed by M. Saghaei, MD, Isfahan University of Medical Science, Isfahan, Iran) using the block randomization method developed by a research fellow at Seoul National University Hospital who is not involved in our trial. The patients who meet the selection criteria will be randomized at a 1:1 ratio in each group (test or control). As this is a double-blinded study, the researchers, patients, and other study personnel will be blinded to the treatment.

### Interventions and management of the study drug

The enrolled patients will be randomly assigned to the test group or the control group. The drug administration will start within 30 days of successful placement of the index ERBD with a SEMS. The specifications of the stent (covered/partially covered/uncovered, caliber, length) were also decided by the physician’s discretion, taking into account the feature of obstruction site and the patient’s condition. And we allow the use of all biliary SEMS available in each institution regardless of manufacturers including the WallFlex biliary stent (Boston Scientific, Natick, MA, USA), the Bonastent (Standard SciTech Inc., Seoul, South Korea), the Niti-S biliary stent (Taewoong Medical Co., Ltd., Ilsan, South Korea), the Zilver biliary self-expanding stent (Wilson-Cook Medical Inc., Winston Salem, NC, USA), and the ARISTENT (CGbio Co., Ltd., Seongnam-si, South Korea), etc. Prophylactic rectally administered indomethacin or rectally administered diclofenac for post-procedural pancreatitis will not be used because none of the rectally administered non-steroidal anti-inflammatory drugs is commercially available in Korea. The patients in the test group will take one 100-mg enteric-coated aspirin tablet (Daewon Pharmaceutical Co., Ltd., Seoul, South Korea) per day before or after meals for 6 months, which was known to be physiologically equivalent to 75 mg plain aspirin [20]. And the patients in the control group will take one placebo tablet for 6 months. We planned a longer period of aspirin use for 6 months in comparison with the previous retrospective study [11] because the known median patency of SEMS in distal CBD obstruction was over 6 months [1, 5]. The patients will be followed-up in an outpatient clinic irrespective of whether the clinical outcomes, including stent malfunction, occur. Anti-cancer therapy, which is or will be received, will continue regardless of whether the patient is enrolled in the study.

The initial drug distribution and storage will be handled by the clinical trials center pharmacy of Seoul National University Hospital. Thirty-five tablets will be packaged in each medicine bottle, which will have a random number on the front label. After screening and enrollment, a randomization number will be assigned to each patient, and a prescribed number of medications will be given to them based on the randomization number. Each patient will be prescribed one bottle every 4 weeks according to the follow-up schedule, and the number of drugs remaining at the following outpatient visit will be retrieved to assess patient compliance. A research nurse will conduct frequent telephone monitoring to be prepared for the cases that lose or do not return the drug bottle.

During the follow-up period, the study patients will be instructed not to take medicines that are prohibited in conjunction with aspirin according to the pharmaceutical authorization from the Korean Ministry of Food and Drug Safety. When taking new medicines during the study period, the patients will be required to contact the researchers so that they can evaluate the drugs’ interactions with aspirin and effect on the research sustainability. The detailed principles of the concomitant use of drugs in this study are as follows:

1. Anticoagulants, thrombolytics, other platelet-aggregation inhibitors, hemostatic agents: de-escalation of dose or careful administration
2. Other non-steroidal anti-inflammatory drugs and salicylic acid preparations: discontinue administration if possible, do not concomitant use due to increased risk of bleeding or decreased renal function
3. High-dose methotrexate over 15 mg/week: do not use due to increment of toxicity
4. Lithium: careful administration due to the possibility of lithium poisoning
5. Selective serotonin-reuptake inhibitors: careful administration due to increased risk of upper gastrointestinal bleeding
6. Digoxin: careful administration due to increment of plasma digoxin level
7. Valproic acid: careful administration due to increment of toxicity
8. Alcohol: careful use due to increment of gastrointestinal mucosal damage, prolonged bleeding time

### Study outcome and assessment

The primary outcome of this study is the rate of stent dysfunction at 6 months. Stent dysfunction is defined as the presence of symptoms of obstructive jaundice or cholangitis in combination with confirmation of stent obstruction or migration via imaging (i.e., computed tomography and/or magnetic resonance imaging) after index SEMS’ insertion. Cholangitis will be diagnosed according to the Tokyo Guideline 2013 as follows: fever over 38 °C, evidence of an inflammatory response with an abnormal white blood cell level (< 4000/uL or > 10,000/uL) or a C-reactive protein level ≥ 1 mg/dL, jaundice (total bilirubin ≥2 mg/dL), abnormal liver function tests (alkaline phosphatase, gamma-glutamyl transferase, alanine transaminase, aspartate transaminase > 1.5 x standard deviation), and biliary dilatation at imaging tests [21]. The rate of stent dysfunction is defined as the percentage of patients who develop stent dysfunction during the follow-up period.

The secondary outcomes of this study are the duration of stent patency, the rate of reintervention, and any adverse events related to aspirin administration. The duration of stent patency will be evaluated on a day-scale as the duration between the index SEMS’ placement and the occurrence of stent dysfunction, or death in the case of patients without stent dysfunction. The rate of reintervention is defined as the percentage of patients who underwent an additional intervention for biliary drainage during the follow-up period after the index SEMS’ placement. For adverse events, a thorough investigation will be conducted to determine whether the adverse effects were related to aspirin administration.

Baseline characteristics and medical information of patients are also recorded in detail through the case report form including information as follows for further analysis: etiology, stage of disease, histologic feature, Charlson’s comorbidity index, history of hypertension, diabetes mellitus, pulmonary tuberculosis, cerebrovascular disease, coronary artery disease, liver or bile duct disease, peptic ulcer disease, any bleeding event, drugs in current use, smoking, alcohol use, family history, prior management including sphincterotomy, whether any biliary stent, further management including detailed information of chemotherapy, radiotherapy, surgery.

The patients will visit the outpatient clinic at 4, 12, and 24 weeks after index ERBD where their clinical symptoms will be evaluated and laboratory tests conducted to assess their vital signs, the presence of Charcot’s triad (jaundice, fever, and right upper quadrant abdominal pain), white blood cell count, hemoglobin, platelets, blood urea nitrogen, creatinine, total bilirubin, alkaline phosphatase, alanine transaminase, aspartate transaminase, C-reactive protein, prothrombin time, and the results of imaging tests such as computed tomography, magnetic resonance imaging, or endoscopic ultrasonography. Additional visits to the outpatient clinic or emergency room at the follow-up center will be allowed if worrisome symptoms or signs become apparent.

### Safety and adverse events

An adverse event is an undesirable medical event that occurs in a patient who is receiving a medication or is being studied, irrespective of whether the event is related to the treatment. In our study, any adverse events, regardless of the seriousness, underlying disease, or association with the test drug, will be noted on the appropriate page of the case record. The classification of the adverse events and their severity will be evaluated according to the National Cancer Institute Common Toxicity Criteria (version 3.0) [22] and will be described in the case record in detail. The causality between any adverse reactions and the intervention will be determined based on the judgment of the researchers. If any unanticipated adverse events related to the research, these will be reported to the Institutional Review Board.

The bleeding that occurs after taking aspirin is the most commonly expected adverse event. However, we believe that major bleeding will rarely occur within 6 months of aspirin use in this study because a recently published meta-analysis reported that major gastrointestinal bleeding or hemorrhagic stroke events occurred at a rate of only 1.71 per 1000 person-years of low-dose aspirin exposure as primary prevention in hemorrhagic stroke patients [23]. We anticipated that the expected effect of aspirin on inhibiting stent dysfunction would be of sufficient benefit to patients from this inference. Also, we tried to exclude cases with a higher risk of bleeding due to aspirin use via the exclusion criteria. In addition, endoscopic biliary stenting is known as a low-risk procedure in the ASGE guidelines [24]. Whether to add a proton-pump inhibitor is decided at the discretion of the physician when necessary.

### Sample size

We calculated the sample size for this study based on the results of a previous retrospective study with a similar clinical situation and purpose [11]. In the study, stent occlusion occurred in 15.3% of the patients in the aspirin group and 23.4% of the patients in the non-aspirin group at a mean of 3 months during the follow-up period. Based on the findings of other studies, we assumed a 40% stent occlusion rate of SEMS in malignant distal CBD obstruction after 6 months [9, 25–27]. Furthermore, we assumed the rate of stent dysfunction on the basis of a recent study that reported the hazard ratio (0.49; 95% confidence interval, 0.32–0.75) was lower in the patients in the aspirin-test group as opposed to 20% in the non-aspirin group [11]. Our calculations were performed using 80% power and a two-sided 5% significance level to verify the null hypothesis and the alternative hypothesis. The sample size required to demonstrate this was calculated as 82 patients in each group and 184 patients in the total sample (92 patients per 10% dropout rate).

### Data analysis

Blinding will remain in place until the statistician codes the statistical analyses of the primary and secondary outcomes. The statistical analyses will be done using the full analysis set according to the intention-to-treat principle, meaning that all the randomized patients will be analyzed in their allocated groups regardless of any protocol violations or early treatment discontinuations. We will also evaluate the outcomes through a per-protocol analysis set that will consider only the subjects who followed the protocol effectively. No formal interim analysis is planned.

We will compare the rate of stent dysfunction using Pearson’s chi-squared test with Fisher’s exact test and calculate the odds ratio of the event. The secondary outcomes (i.e., the duration of stent patency, the rate of reintervention, and the adverse events related to aspirin administration) will be analyzed using Pearson’s chi-squared test with Fisher’s exact test, Student’s *t* test and Kaplan-Meier curves, stratified by drug, and the hazard ratios between two groups measured using the Cox proportional hazards regression. We also plan to identify the further affecting factors for stent dysfunction by univariate and multivariate logistic regression analysis.

### Quality assurance and control

The research institutes will be monitored centrally and via on-site visits by a research nurse from Seoul National University Hospital who will follow the ICH-GCP guidelines. The trial institutes will also be visited regularly by the study coordinator and the study sponsor to ensure compliance with the study protocol and ICH-GCP guidelines. Complete source data verification will be performed by independent monitors. All the data collected during the trial will be stored in a secure electronic data capture system according to the ICH-GCP guidelines. A central study coordinator will coordinate the study. The Seoul National University Hospital will monitor the study’s progress and quality and the completeness of the study data.

### Access to data

The principal investigators and co-investigators will have access to the final trial dataset.

### Dissemination of data

The results of this study will be disseminated via professional journals and national, and international conferences. A lay summary of study results will be provided with ClinicalTrials.gov upon study completion.

### Protocol modifications

Major protocol modifications are not anticipated. If any important modifications occur, they will be notified through a modification request form to the Institutional Review Board of Seoul National University Hospital and communicated to all study personnel.

### Authorship

Authorship will be given to those who make considerable contributions to the study.

## Discussion

Our study is the first randomized controlled clinical trial on the efficacy of aspirin in patients with SEMS for malignant distal CBD obstruction. Endoscopic SEMS’ placement is currently regarded as the standard palliative treatment for patients with unresectable, malignant distal CBD obstruction. SEMS have a larger luminal diameter and guarantee longer patency than plastic stents, but their benefits in terms of survival have not been established despite several meta-analyses on the subject [1, 28, 29]. Nevertheless, longer stent patency may be important to maintain quality of life during the survival period post SEMS’ placement for patients with malignant distal CBD obstruction and to reduce the need for reintervention or the potential for cholangitis since the expected patient life span is increasing due to the development of palliative treatments, including chemotherapy, immunotherapy, and radiotherapy. Recently, the impact of biliary-stent-related adverse events during palliative chemotherapy has been emphasized from the perspective of patient morbidity [30].

Research trends show that physicians are mainly focused on the mechanical aspects of SEMS to increase patency, and the structure and composition of these stents are constantly evolving [31–34]. Despite this, the need for improved stent patency has not been met because of the lack of research on chemical prophylaxis for stent patency. Based on the study of Jang et al. [11], we will investigate whether aspirin use after index ERBD with SEMS for malignant distal CBD obstruction is effective and safe. Our study may provide further understanding of the potential role of aspirin in this patient population. If aspirin can help to maintain stent patency, patients will be able to keep their stents for longer periods of time alongside the very simple administration of one tablet a day, and this will reduce the need for reinterventions. Because aspirin is easily available, cheap, and safe with relatively few side effects, it can have a significant impact if it is found to be effective in this well-designed prospective study.

For this study, we have chosen to use aspirin as a chemoprophylactic agent to improve stent patency for several reasons. First, aspirin is considered to be very safe because it has been used by countless patients for a long time, and its efficacy, mechanism of action, and side effects have been studied comprehensively. Even though, there has been debate as to whether it actually reduces gastric-mucosal damage. Second, the anti-inflammatory effects of aspirin that inhibit the formation of prostaglandin by COX-2 inhibition seem to reduce the tissue inflammation induced by SEMS. Third, the use of aspirin may inhibit both mucin formation in the gallbladder, which is associated with biliary stone formation, and the coating with mucinous bile of the stent that may affect stent patency [14–17]. Nevertheless, the results of some studies have shown that the effects of aspirin are unclear. Last, the researchers of several basic and clinical studies have suggested that aspirin has an anti-tumor effect, and some large epidemiological studies, clinical studies, and meta-analyses have indicated that aspirin may have a significant effect on the prevention and treatment of cancer, especially colon cancer [35–40]. Several basic studies at various molecular genetic levels have provided an explanation for the anti-tumor effect of aspirin. It is known that aspirin leads to cell apoptosis by inhibiting Bcl-2 expression and downregulating COX-2 expression, which is known to convert arachidonic acid to prostaglandins, and it may reduce tumor-cell invasion through the downregulation of MMP-2 expression [41, 42]. Some studies have also provided evidence that aspirin promotes deoxyribonucleic acid (DNA) self-healing by the upregulation of hMLH1, hMSH2, hMSH6, hPMS2 [43], and XRCC expression [44], slowing down gene-mutation accumulation, and protecting normal cell DNA [32, 45]. Recent studies have suggested several new aspirin pathways, such as inhibiting the synthesis of prostaglandin E2 (PGE2) [46], controlling the number of circulating platelets and their activity levels to evade several innate anti-tumor effects [47–50], increasing chemo-agent toxicity, and downregulating cellular drug resistance [51, 52]. Since the results of these basic studies have mostly been conducted in vitro and have not been proven in humans, these outcomes cannot be presented as definitive mechanisms of aspirin’s effects.

This study will have some limitations. First, the patients will have malignant obstruction from various types of malignancies, and they may be receiving different kinds of treatments. This may affect SEMS’ patency. To mitigate this, we will conduct a subgroup analysis of the effects of the different treatments and cancers rather than control such factors because it is our opinion that aspirin could be effective even when prescribed in a variety of clinical situations. Second, we have had to make some assumptions in the calculation of the sample size. We calculated the sample size based on the most reasonable approach, but it is undeniable that this is a logical leap because stent patency has been reported very differently in diverse clinical situations.

The results of this study may provide a new evidence-based treatment option for patients with malignant distal CBD obstruction.

## Trial status

This study is approved as protocol version 1.2 at the Seoul National University Hospital on 4 September 2017. The trial is currently in the recruitment phase. Study enrollment began on 12 October 2017. It is estimated that recruitment will be completed by 12 September 2019, with a study completion date of 5 September 2020.

## Supplementary information

**Additional file 1.** Standard Protocol Items: Recommendations for Interventional Trials (SPIRIT) 2013 Checklist: recommended items to address in a clinical trial protocol and related documents*.

## Data Availability

The datasets generated and/or analyzed during the current study will available from the corresponding author on reasonable request. In addition, following the completion of the study, the authors plan to make data available via publication.

## Abbreviations

AIMS: Aspirin for metal stents in malignant distal common bile duct obstruction
CBD: Common bile duct
ERBD: Endoscopic retrograde biliary drainage
ICH-GCP: International Conference on Harmonization and World Health Organization Good Clinical Practice
SEMS: Self-expandable metal stents
UDCA: Ursodeoxycholic acid
